# Hepatitis C and Metabolic Disorders: Genetics, Mechanism, and Therapies (Clinical and Experimental)

**DOI:** 10.1155/2012/937202

**Published:** 2012-12-13

**Authors:** Mario Reis Alvares-da-Silva, Heiner Wedemeyer, Helena Cortez-Pinto, Claudia Pinto Marques Souza de Oliveira

**Affiliations:** ^1^Gastroenterology Division, Hospital de Clínicas de Porto Alegre, Ramiro Barcelos 2350, 90035-003 Porto Alegre, RS, Brazil; ^2^Department of Hepatology and Endocrinology, School of Medicine, University of Hannover, Hannover, Germany; ^3^Department of Gastroenterology, Institute of Molecular Medicine, School of Medicine, University of Lisbon, Lisbon, Portugal; ^4^Department of Gastroenterology, School of Medicine, University of Sao Paulo, Sao Paulo, SP, Brazil

Hepatitis C is one of the major causes of liver diseases worldwide. The spectrum of the disease is quite variable, ranging from acute hepatitis to cirrhosis and hepatocellular carcinoma. Also, it is one of the main causes of liver transplantation. The natural history starts again after transplantation since hepatitis C virus (HCV) reinfects the new liver in almost all the cases. 

Liver is by far the most often affected organ, but hepatitis C is definitely not a liver-limited disease. Actually, HCV leads to a systemic disease. Mixed cryoglobulinemia, chronic lymphocyte stimulation, chronic kidney disease, insulin resistance, and diabetes mellitus are known extrahepatic manifestations of HCV. Also, the virus has a role in the lipid metabolism, as total serum cholesterol and triglycerides levels are usually low in HCV-infected patients. So it seems worth to discuss the extrahepatic manifestations of the virus, especially the HCV-associated metabolic disturbances. 

This special issue provides a broad review of HCV influence on metabolic and extrahepatic disease, from experimental to clinical research, discussing some of the most important issues in the area. Studies from different countries and universities highlight the HCV worldwide clinical importance. 

A. C. M. Nascimento and colleagues, from Brazil and Australia, focused on indigenous Western Amazonians HCV-infected patients (76.3% genotype 1). In this very particular sample, most of the HCV-infected individuals have body mass index (BMI) lower than 25, but they have more hepatic steatosis and fibrosis than patients with hepatitis B infection from the same region. Interestingly, the presence of steatosis was not correlated with either BMI, arterial hypertension, glycaemia, or serum levels of cholesterol and triglycerides, suggesting a direct viral-induced lesion. 

Insulin resistance is closely related to liver steatosis as extensively demonstrated in nonalcoholic fatty liver disease (NAFLD). Also, HCV itself can lead to insulin resistance, and many studies evaluated this metabolic complication in HCV. However, potential confounders frequently are a problem, as many of those studies included obese patients with diabetes and dyslipidemia. Two studies from Southern Brazil have attempted to investigate insulin resistance in a low-risk virus C infected population (nonobese, noncirrhotic, nondiabetic and nondyslipidemic) and were also published in this special issue. As the results reported in the Amazonian population, people from the south of Brazil, a region characterized by its strong Italian, German, and Portuguese inheritance, displayed more insulin resistance when infected with HCV. C. R. Kappel and colleagues showed that HCV is related to lower IRS-1 levels. IRS-1 and IRS-2 are proteins of a family of ligands and molecules that connect insulin receptors to a cascade of reaction, allowing the entry of glucose into the cell. Thus, it seems that HCV downregulates this receptor in the absence of obesity and other risk factors. Also, in NAFLD, steatosis is associated with low serum levels of adiponectin. Studies on adiponectin levels in hepatitis C often reach contradictory results. In this special issue, M. T. Michalczuk et al., also studying a low-risk population, found no differences between adiponectin serum levels in patients with or without HCV. As insulin resistance was significantly higher in HCV patients and unrelated to the total cholesterol levels, the authors concluded that lipid metabolism probably did not influence this viral-related insulin resistance.

Intestinal microbiome has been increasingly considered important. Indeed, we have more intestinal-derived bacterial than human genes. Recent research is consistent in confirming that the microbiota has a close relationship to the immunological control and also regulates some molecular pathways that culminate in the lipid homeostasis. Currently, microbiota is seen as a metabolic organ, and obesity itself as well as liver steatosis has been related to the dysbiosis of the intestinal microbiome. Studies on the microbiome of HCV patients are lacking, but there are some studies on hepatocellular carcinoma (HCC). In this special issue, a very interesting study from Norway and Australia, conducted by a Nigerian researcher, focused on the influence of Helicobacter bilis on HCC, studying the expression of bacterial-associated proteins in Huh7 cells harboring HCV replicon and also in replicon-cured cells. Okoli and colleagues showed that the differential expression of some tumor-related proteins and HCC-associated genes in Helicobacter-infected cells provided evidence for the carcinogenic effects of *Helicobacter bilis* on the Huh7-derived cell lines. Further studies on intestinal microbiome of HCV patients are needed to clearly establish the influence of the microbiota on HCV natural history and complications.

HCV systemic consequences can occur in other organs. Some neuropsychiatric complications are related to HCV, like depression, and hepatic encephalopathy is a classical complication of cirrhosis. High levels of ammonia play a synergistic role with electrolyte imbalance and neuroinflammation in the pathogenesis of hepatic encephalopathy. Less is known regarding brain disease in noncirrhotic patients. In this special issue, R. Nagarajan and colleagues, from USA, evaluated HCV-associated neurocognitive dysfunction by means of 2D MR brain spectroscopy. Patients with HCV showed significantly increased brain myoinositol and glutathione, indicating a higher brain oxidative stress and inflammation than in controls. If these abnormalities can explain some frequent symptoms, like fatigue, then it is an open research area.

Moreover, N. Y. Akamatsu and Sugarawa, from Japan, made a comprehensive review of HCV in the liver transplant setting. They highlighted some factors associated with the severity of recurrent hepatitis C after transplantation, including genetics. IL28B gene polymorphism can exert influence on the recurrence of the disease, as many studies are showing that patients with non-CC genotypes have a more severe disease.

Finally, M. Enjoji and colleagues, from Japan, have discussed HCV therapy on a metabolic basis. They commented about the ability of HCV in creating a favorable microenvironment into the hepatocyte, by changing the expression of lipid-metabolism-associated factors, conferring to the virus some advantages in its life cycle. They also brought light on the cannabinoid system, discussing the type 1 cannabinoid receptor activation and its influence in liver steatosis by increasing several lipogenic factors, like SREBP-1c and fatty liver synthase, at the same time decreasing intrahepatocyte fatty acid oxidation. They made an interesting review of insulin sensitizers, antioxidants, and statins in chronic hepatitis C.

Thus, this special issue can provide the reader a comprehensive overview on HCV-related metabolic disorders, although not covering every aspect of the topic.

*Mario Reis Alvares-da-Silva* *Mario Reis Alvares-da-Silva* *Heiner Wedemeyer* *Heiner Wedemeyer* *Helena Cortez-Pinto* *Helena Cortez-Pinto* *Claudia Pinto Marques Souza de Oliveira* *Claudia Pinto Marques Souza de Oliveira*

